# The GF-3 SAR Data Processor

**DOI:** 10.3390/s18030835

**Published:** 2018-03-10

**Authors:** Bing Han, Chibiao Ding, Lihua Zhong, Jiayin Liu, Xiaolan Qiu, Yuxin Hu, Bin Lei

**Affiliations:** 1 Key Laboratory of Technology in Geo-Spatial Information Processing and Application Systems, Chinese Academy of Sciences, Beijing 100190, China; cbding@mail.ie.ac.cn (C.D.); lhzhong@mail.ie.ac.cn (L.Z.); liujy@mail.ie.ac.cn (J.L.); xlqiu@mail.ie.ac.cn (X.Q.); yxhu@mail.ie.ac.cn (Y.H.); leibin@mail.ie.ac.cn (B.L.); 2 Institute of Electronics, Chinese Academy of Sciences, Beijing 100190, China; 3 National Key Laboratory of Microwave Imaging Technology, Beijing 100190, China

**Keywords:** Gaofen-3, SAR, data processor, system architecture, processing algorithm, format of products

## Abstract

The Gaofen-3 (GF-3) data processor was developed as a workstation-based GF-3 synthetic aperture radar (SAR) data processing system. The processor consists of two vital subsystems of the GF-3 ground segment, which are referred to as data ingesting subsystem (DIS) and product generation subsystem (PGS). The primary purpose of DIS is to record and catalogue GF-3 raw data with a transferring format, and PGS is to produce slant range or geocoded imagery from the signal data. This paper presents a brief introduction of the GF-3 data processor, including descriptions of the system architecture, the processing algorithms and its output format.

## 1. Introduction

Gaofen-3 (GF-3) is a Chinese spacecraft carrying a C-band SAR (5.4 GHz), which was launched in August 2016, from Taiyuan (Shanxi Province, China). The GF-3 satellite went through a four-month payload performance commissioning phase and a two-month application performance commissioning phase, and from January 2017 began to provide customers with advanced, commercially-available spaceborne SAR imagery which had fully polarization mode and resolution as fine as 1 m in spotlight mode [1]. The GF-3 system is able to generate a greater diversity of data products than any other civilian satellite SAR in China [2]. Besides single polarization stripmap and scanSAR mode as in HuanJing-1C (HJ-1C) SAR [3], GF-3 SAR can also operate at high resolution spotlight mode, dual-receive stripmap mode, dual polarization stripmap or scanSAR mode, and quad polarization stripmap mode, which can be separated into 12 observing modes by different resolution and swath [1].

The GF-3 data processor (GF3DP) is the crucial part within the whole GF-3 ground segment, and it was developed by Institute of Electronics, Chinese Academy of Sciences (IECAS) as a promotion to the original IECAS SAR data processing system, which supports the processing of various data products for other preceding SAR satellites in China. The GF3DP consists of two subsystems of the GF-3 ground segment, which are referred to as the data ingesting subsystem (DIS) and the product generation subsystem (PGS) physically located at China Center for Resources Satellite Data and Application. The DIS is to record and catalogue GF-3 raw data in transferring format from the receiving stations, and the PGS is to produce slant range or geocoded imagery from the SAR signal data of all observing modes.

This paper will give a brief introduction of the architecture, the processing algorithms and the output format of GF3DP. Typical processing results both at the commissioning phase and after GF-3 satellite has been in operational application will also be provided in the later section.

## 2. System Architecture

The GF-3 ground segment, which is composed of the operational management subsystem (OMS), the data receiving subsystem (DRS), the data ingesting subsystem (DIS), the product generation subsystem (PGS), the image quality subsystem (IQS), the data archive and information management subsystem (DIMS) and the data distribution subsystem (DDS), applies a Linux^®^/PC combined hardware architecture, which integrates the power, scalability, and reliability of the Linux platform with the flexible operation of the PC. The control and monitor for the processing are executed on the operator workstations with standard Windows^®^/Intel^®^PCs, but the whole GF3DP including DIS and PGS runs on Intel^®^Xeon^®^ CPU E5-2670 (Intel, Santa Clara, CA, USA) servers with 16 processors and 96 GB of RAM, which have Red Hat Enterprise Linux Server release 6.4 as the platform. The Linux servers were separated into two groups, one group was used to ingesting the raw data, and the other group was used to generate image products. More than 20 servers are continuously running every day to support the daily throughput and product loads of the GF-3 ground segment. The number can be easily increased for the scalability of the overall system to meet a greater required capacity in the future. In addition, the communications among DIS or PGS with other subsystems are mainly completed via Web Services.

**Figure 1:**
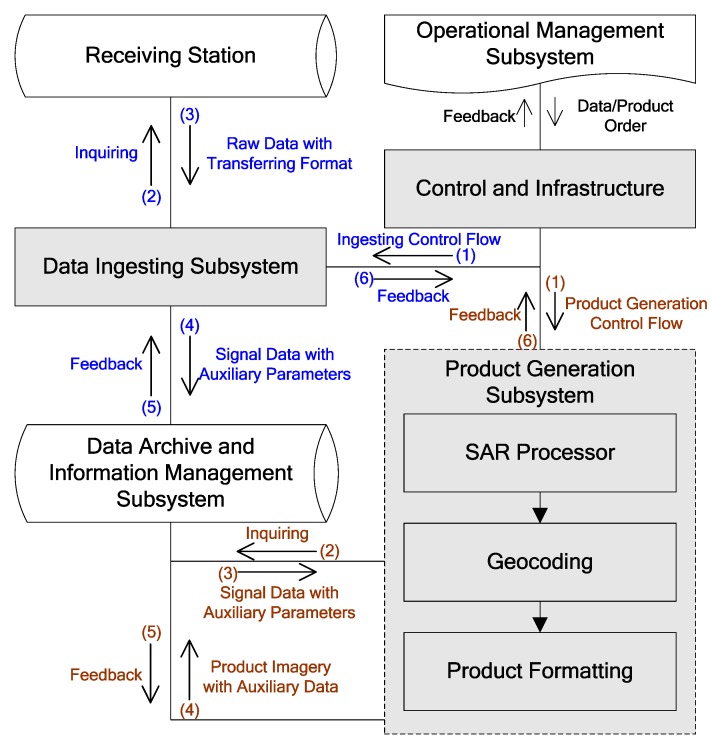
GF3DP software architecture.

The GF3DP software is based on the IECAS multi-mission data processing architecture. It was reconstructed to adapt for Gaofen series interfaces and upgrades the data processing system to perform raw data ingesting and product generation of GF-3 besides the currently supported full resolution quick-viewing of HJ-1C and SAR data processing of other remote sensing satellites of China. The GF3DP software is composed of several components which are filled with gray color in Figure 1.

### 2.1. Control and Infrastructure

The GF3DP operation is controlled by work orders as indicated in Figure 1. According to the task, the orders received by GF3DP are divided into two types, namely the data orders and the product orders which are both sent by OMS. The data order was first validated by the work order controller of GF3DP, basing on which the corresponding work order sent to DIS will be created. This work order extracted from data order is used to start the raw data ingesting process, and there will be a feedback about the results of ingesting sent by DIS. The control of data ingesting is indicated by blue color in Figure 1. After getting the feedback on that the raw data has been processed and archived, OMS will send a product order to GF3DP to generate image product for every logical scene created in the data ingesting process. For each product order that the GF3DP receives from OMS, the work order controller will validate both the format and content first, and then creates corresponding work order which will be sent to PGS. This work order received by PGS is used to start the product generation process. After the required product has been successfully generated and archived, the GF3DP will respond to the OMS, sending a feedback about the processing results. The control of product generation is indicated by brown color in Figure 1. The GF3DP supports multiple concurrent processing tasks by using multiple Linux servers. The GF3DP also allows the operator to create, modify, execute, stop, and reject work orders as needed.

### 2.2. Ingesting

The DIS of GF3DP is responsible for ingesting GF-3 raw data sent by DRS of GF-3 ground segment, which consists of two major software components: the transferring-format decoding and quick viewing software, and the logical-scene generating and data cataloging software. First, DIS extracts SAR data from the downlink by decoding the transferring-formatted signal. After the auxiliary data of the SAR echoes being validated and analyzed, DIS will sort the SAR raw data into a suitable format for the SAR processor of PGS, and it will also extract platform parameters, sensor parameters and all other parameters may be needed in the succeeding process. At the same time, a quick viewing of the echo data will be performed to check the status of the sensor and the quality of the echo signal. Second, each sorted data will be logically divided into several scenes to keep every final product image approaching a square on the map, but it will not be physically separated into different data files. Finally, after the echo data file and auxiliary data files are generated, DIS will ask DIMS for archiving task through Web Services.

### 2.3. SAR Processor

The SAR processor is the critical software component of PGS, which includes three major software modules: the stripmap processor, the spotlight processor and the scanSAR processor. The SAR processor mainly performs SAR echo data decoding, Doppler parameters calculating, radiometric corrections and focusing the signal data into output imagery.

The stripmap processor is responsible for the imaging processing of ultra-fine stripmap mode, fine stripmap mode, wide fine stripmap mode, standard stripmap mode, quad-pol stripmap mode, wide quad-pol stripmap mode, wave mode and expanded incidence angle mode of the GF-3 satellite, which employs the chirp scaling (CS) algorithm [4] as a basic algorithm. Some supplementary have been applied to the basic processor to support the accurate focusing and multi-mode processing, especially for ultra-fine stripmap mode and quad polarization stripmap mode, about which there will be a brief description in the later section.

The spotlight processor is responsible for the imaging processing of spotlight mode of the GF-3 satellite, namely, it performs focusing of routine sliding spotlight signal, but its processing setting is not suitable for staring spotlight imaging. The spotlight processor employs the deramped chirp scaling (DCS) algorithm [5,6] as a basic algorithm, but several enhancements have been applied to the basic processor to support focusing of GF-3 1 m resolution spotlight SAR imaging, which will also be described in detail in later section of this paper.

The scanSAR processor is responsible for the imaging processing of narrow scanSAR mode, wide scanSAR mode and global observation mode of GF-3 satellite. It uses the extended chirp scaling (ECS) algorithm [7] as a basic algorithm, which completes azimuth compression basing on “deramping” and is quite suitable for scanSAR imaging with burst-mode SAR data. Some enhancements on antenna pattern correction have also been applied to the basic processor to suppress the scalloping of GF-3 scanSAR mode.

Each SAR processor above accepts the data with ingesting format by DIS as the input, and its output imagery has been radiometric corrected [8], which is in slant range geometry and may be single look or multiple looks depending on the setting of the product order received from OMS. All these processors use the architecture of parallel processing, and the image throughput can be easy increased by adding Linux servers to the GF-3 ground segment.

### 2.4. Geocoding

The geocoding processor of GF3DP either executes a standard map projection for the imagery in slant range geometry to a uniform earth-fixed grid, or only generates a rational polynomial coefficient (RPC) file corresponding to the map projection process. Inherent geometric distortion in the SAR data caused by the side looking geometry, surface terrain, system time error, and platform velocity variation have been all considered in GF-3 SAR image geocoding [9]. Here the pixel locations are derived by using range-Doppler model for the sensor imaging geometry and the target elevation. The GF3DP only supports geocoding of products using orbit data from downlink and average digital elevation in a scene from SRTM 30 m digital elevation model (DEM) database, but without any ground control points (GCPs).

### 2.5. Product Formatting

As indicated in Table 1, GF3DP formats the single look complex imagery (SLC) or single/multiple looks intensity imagery (SLI/MLI) in slant range geometry, namely the Level 1A or Level 1B product, as TIFF imagery with corresponding RPC file, and the geocoded imagery (SGC), namely Level 2 product, as GeoTIFF imagery.

## 3. SAR Algorithm Design

### 3.1. High-Precision Processing of Spotlight Mode

**Figure 2:**
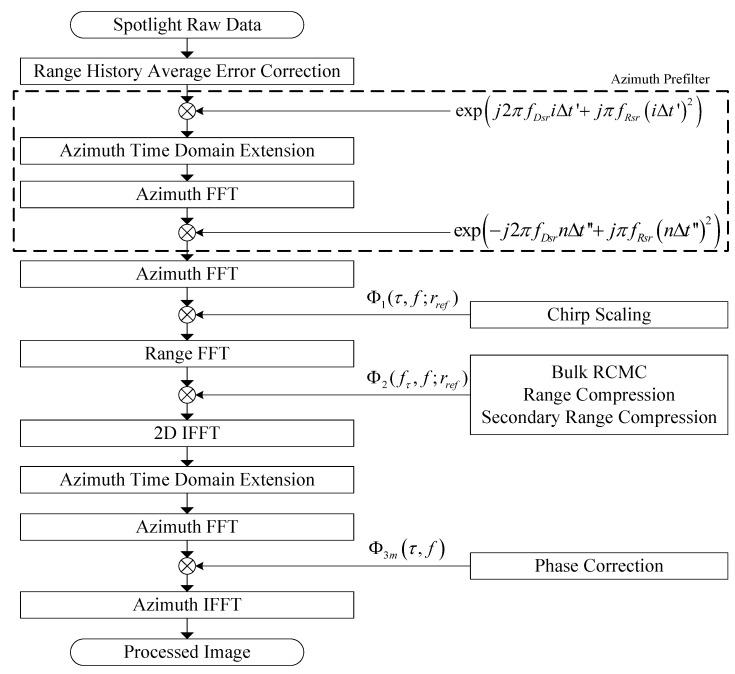
Block diagram of deramped chirp scaling (DCS) algorithm for GF-3 spotlight SAR processing with range history error correction.

GF-3 SAR operates on the sliding spotlight or hybrid stripmap/spotlight mode as a routine spotlight mode, and worked on the staring spotlight mode for only several times after launching as an experimental spotlight mode. The spotlight processor of GF-3 employs the DCS algorithm [5,6] as a basic processing algorithm, which is mainly used for data processing of the routine spotlight mode. Concerning on the long imaging time and long synthetic aperture of high-resolution (1 m) and wide-swath (10 km) spaceborne spotlight SAR of GF-3, several enhancements to improve the processing precision and to adapt for the azimuth-space-variant characteristic of GF-3 spotlight imaging are applied in the processor [10], which have been indicated in Figure 2.

First, motion compensation referred as first-order MOCO in airborne SAR [11] is applied on the GF-3 spotlight SAR signal data before being putted into DCS processor, which is used to correct the azimuth-space-variant error among targets at different sites along azimuth. The motion error to be compensated is indicated as Δ*R_sr_* which describes the non-linear and non-uniform movement of the spaceborne SAR relative to the virtual rotating point [10], which is an average error of the whole scene.

Second, an azimuth prefilter to resolve the aliasing in Doppler domain, which was defined according to [5,10], is applied on the radar echo signal before being putted into the chirp scaling imaging process.

Third, after being azimuth prefiltered, the new azimuth sampling rate will resolve the Doppler aliasing of the signal, and the new azimuth processing number usually has the same order as the original one. Except a small change in the third factor Φ_3_ of chirp scaling algorithm which is required in order to correct for the azimuth prefiltering, the succeeding processing of sliding spotlight data will be almost as the same as stripmap data.

Finally, a method to compensate the remnant cubic phase error ΔΦ*_c_* within the accumulating time is also applied in the third factor of chirp scaling algorithm. The modified Φ_3_ was defined as Φ_3*m*_ according to [10].

The Δ*R_sr_*, ΔΦ*_c_* and Φ_3*m*_ mentioned above will be described in Appendix A.

### 3.2. Azimuth Processing of Dual-Receive Stripmap Mode

**Figure 3:**
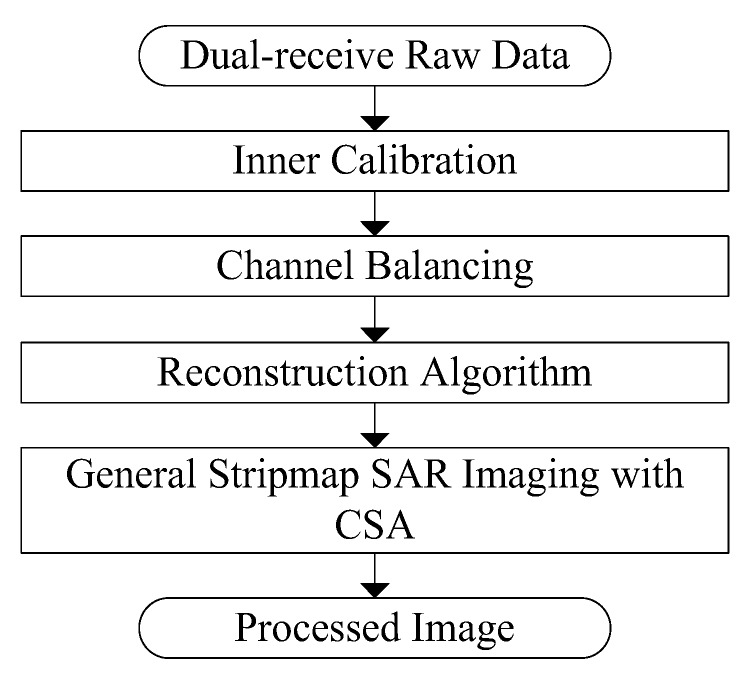
Block diagram of azimuth processing for dual-receiving data of GF-3 ultra-fine stripmap mode.

The GF-3 ultra-fine stripmap mode uses the dual-receive technique to obtain 3 m resolution and 30 km swath simultaneously, which is exactly the same as RadarSat-2 [12]. The samples of dual-receive channels were interleaved by azimuth processing indicated in Figure 3. After this step, a general SAR imaging algorithm for all other stripmap modes can be performed.

First, based on the calibrating data from inner calibration system, the corrections for such as gain, bias, quadrature departure and timing imbalances between dual-receive channels are performed on the raw data.

Second, an imbalance estimating of the two channels is employed to eliminate the residual mismatches error for both amplitude and phase [13].

Third, a reconstruction filter to suppress the azimuth ambiguity from the linear system of equations with respect to the antenna geometry and the SAR parameters is applied here [14].

Finally, after reconstructing of the dual-receive signals from the separated fore- and aft-channels, a general stripmap SAR imaging with CS algorithm is introduced as in all other stripmap observing modes.

An integrated imaging scheme for unambiguous imaging of both static scenes and moving targets was proposed in [15].

### 3.3. Quad-Pol Processing of Quad Polarization Stripmap Mode

**Figure 4:**
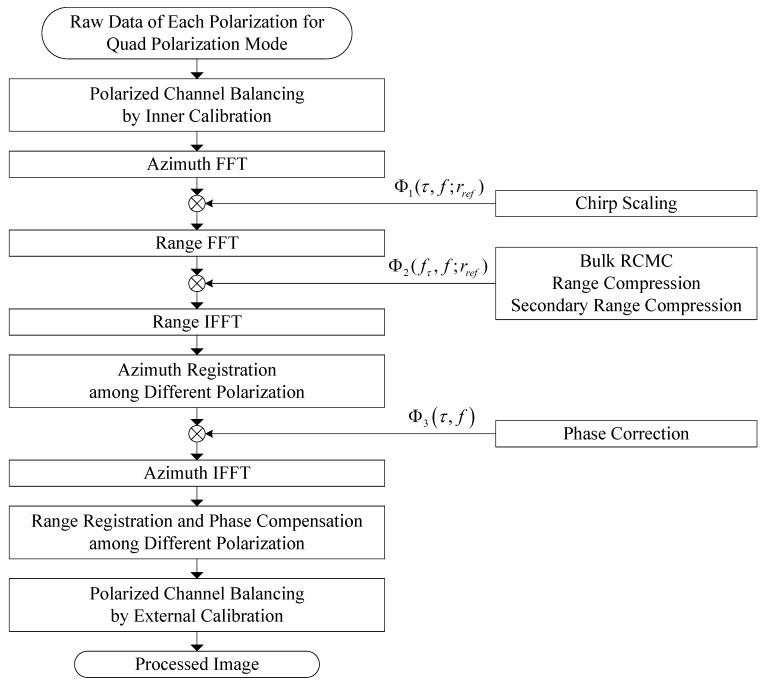
Block diagram of chirp scaling (CS) algorithm for GF-3 quad polarization SAR processing with polarized calibration.

The GF-3 stripmap data can be collected in quad polarization mode by alternatively transmitting horizontal (H) and vertical (V) polarized pulses and simultaneously receiving the H and V polarized echoes from both H and V transmitted pulses. Each polarization signals will be processed sequentially once the quad-pol product order is received by PGS of GF3DP. The four images of the quad-pol mode are generated on the same grid of pixel positions, so each pixel has four values, corresponding to the HH, HV, VH, and VV signals. Figure 4 provides a detailed description of the GF-3 quad polarization SAR processing.

First, based on the calibration data from the inner calibration system, the corrections for such as gain, bias, quadrature departure and timing imbalances among four qual-pol channels are performed on the raw data.

Second, azimuth registration between different transmitting polarization imagery is applied in Doppler domain before azimuth compression. The value of azimuth shifting for registration is just one pulse repeat interval.

Third, due to the space-variant characteristic of the channel imbalance introduced by antenna phase center changing at alternating transmissions and separate receptions, range registration and phase compensation among different polarization are applied on single look complex imagery.

Finally, the remnant systematic imbalances among qual-pol imagery are calibrated basing on external calibration parameters.

### 3.4. Scalloping Suppressing of ScanSAR Mode

**Figure 5:**
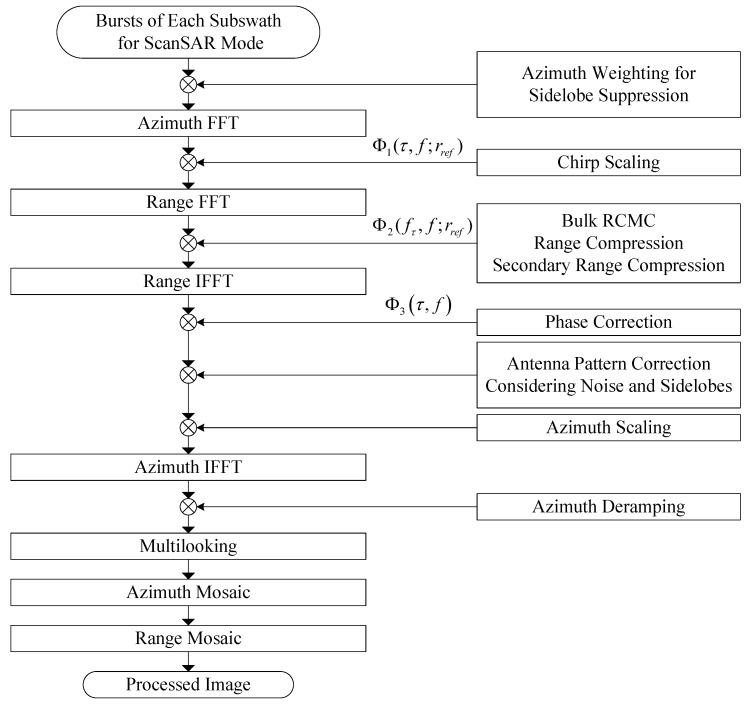
Block diagram of extended chirp scaling (ECS) algorithm for GF-3 scanSAR mode.

The GF-3 scanSAR data processing is performed using the ECS algorithm [7]. There is an azimuth subaperture approach being introduced into the original chirp scaling algorithm for scanSAR image processing, which has been indicated in Figure 5. The azimuth compression is performed by means of the spectral analysis (SPECAN) which has some deficiencies and approximations needed to be overcome for accuracy focusing. In this method, an azimuth scaling function is applied to remove the variation of azimuth frequency modulation with range, which will also bring a range invariant, purely linear frequency modulation. In addition, to suppress the scalloping of scanSAR image, the Doppler centroid estimating and the antenna gain correcting are the crucial part of scanSAR processor. In GF3DP, antenna pattern correction has considered the power of both the system noise and the echoes from the antenna’s sidelobes, which mainly bases on the approach presented in [16].

## 4. Gaofen-3 Product Formatting

As outlined in Table 1, GF-3 products generated by GF3DP can be divided into two levels, namely the Level 1 products which are the imagery in slant range geometry, and the Level 2 products which are the imagery having been geocoded. All levels standard products will consist of single or multiple image pixel data files and a corresponding product-describing file, namely the meta-data file.

### 4.1. Image Pixel Data Files

According to the observing mode of GF-3, the product imagery except the SLC of scanSAR includes one, two, or four image pixel data files, which correspond to the polarimetric operation mode of single, dual, or quad polarization. For each polarization of scanSAR mode, the single look complex image pixel data files were given as imagery of a number of strips, and the numbers for narrow scanSAR mode, wide scanSAR mode and global observation mode are three, five and seven respectively. The format of all image pixel data files is TIFF with RPC file for Level 1 product, and GeoTIFF for Level 2 products. Especially for Level 1 product, the difference between Level 1A product and Level 1B product are two channel TIFF or only one channel TIFF, and the two channels of Level 1A image pixel data file correspond to the real part and the imaginary part of the SLC.

### 4.2. Meta-Data File

The associated information about the GF-3 product is provided as a meta-data file with XML format. The meta-data file of GF-3 product is created as several hierarchical and logical record structures. At the first level, the meta-data are divided into five primary records which consist of “sensor”, “platform”, “productinfo”, “imageinfo”, and “processinfo”. Each primary record is divided into further subrecords, and the detailed description for every subrecord has been given in the GF-3 products description handbook [17] which only has a Chinese version until now. There will be a brief description for the critical records of the meta-data in Appendix B.

### 4.3. Product Delivery

At the final step of the product generation, all the image pixel data files and the associated file will be packed as a “.tar” file, archived by DIMS and automatically delivered to the customer using the file transfer protocol (FTP) by DDS.

## 5. Results

### 5.1. Spotlight Product Focusing Quality Evaluation

**Figure 6:**
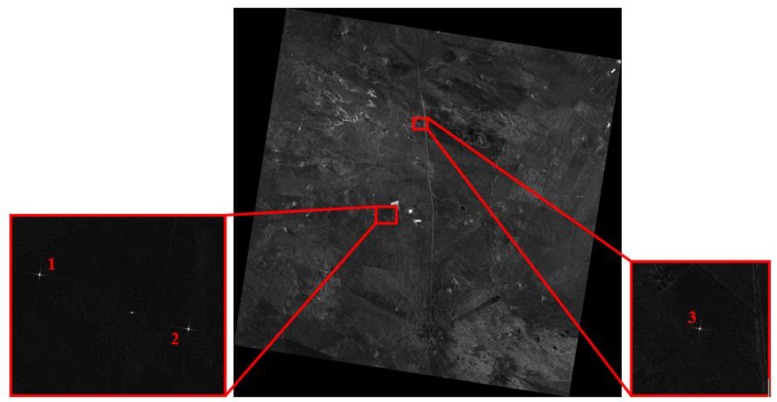
Geocoded product imagery of the performance test site for spotlight mode of GF-3 (The number 1, 2 and 3 indicate the positions of the corner reflectors).

Table 2 presents the primary parameters of one of the performance test experiments for spotlight mode of GF-3 at the commissioning phase. There were three corner reflectors in the experiment as indicated in Figure 6. Corner reflectors No. 1 and No. 2 were placed near the center of the scene, and corner reflector No. 3 was placed in the middle of the center and the edge.

**Figure 7:**
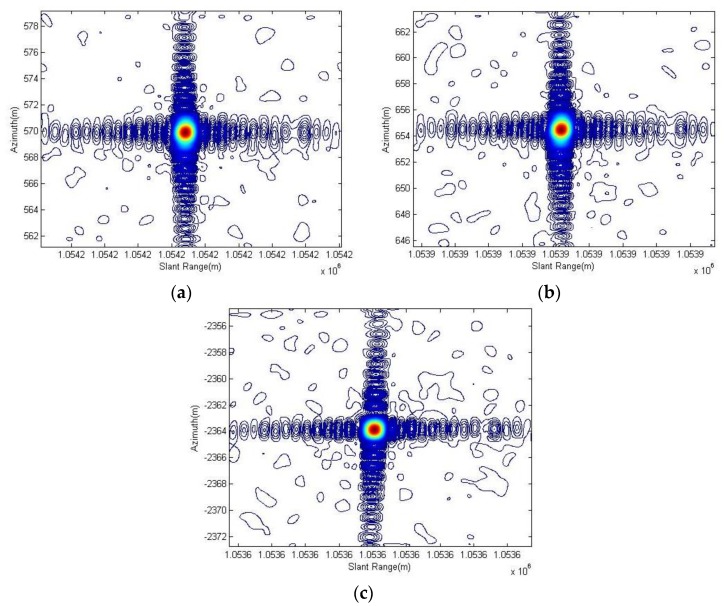
The contour images of corner reflector No. 1, corner reflector No. 2 and corner reflector No. 3 are shown in (**a**–**c**) respectively. These were given before the spotlight processor applied the enhancements.

**Figure 8:**
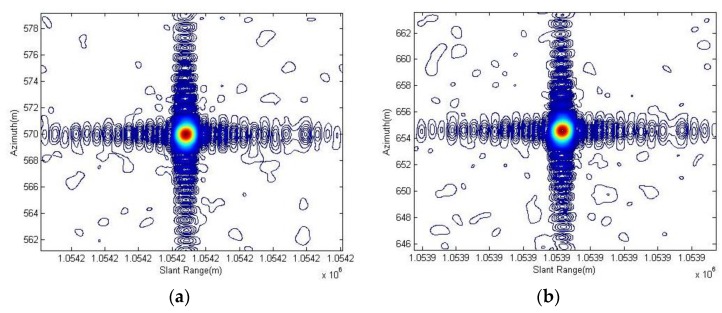

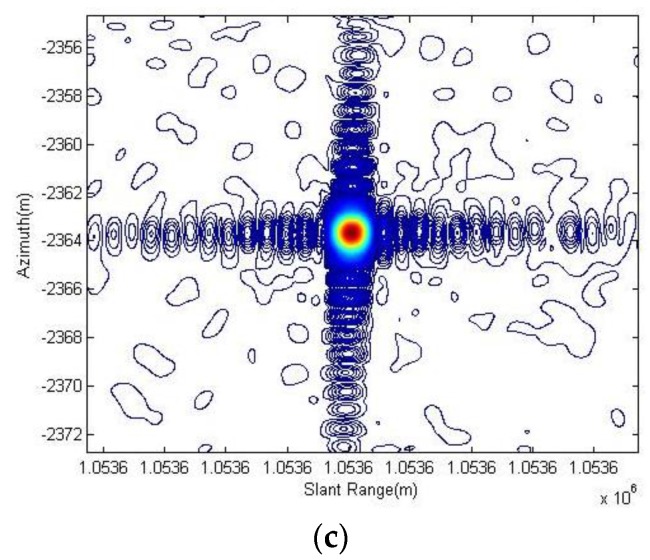
The contours of corner reflector No. 1, corner reflector No. 2 and corner reflector No. 3 are shown in (**a**–**c**) respectively. These were given after the spotlight processor applied the enhancements.

As outlined in Table 3 and Table 4, the resolution, PSLR and ISLR are tested using all three corner reflectors. The results are better at some extent after the spotlight processor applies the motion compensation including both the range history average error correction and the remnant cubic error compensation on the imaging process which are described in Section 3.1, especially for the uniformity of performance among different corner reflectors. Considering the corner reflector No. 3, it presents deterioration in azimuth resolution but gives better PSLR and ISLR after the processing, which is mainly caused by different weighting shapes for Doppler with motion error or not. Figure 7 and Figure 8 present the contour images of the corner reflectors in the product imagery of Level 1A.

Performance test experiments for spotlight mode at different looking angles were carried out for several times at the commissioning phase. Table 5 outlines the performances (in the statistical sense) of azimuth compression at typical center looking angles of 22.37° (left-looking with azimuth scanning angle of ±1.9°), 36.52° (right-looking with azimuth scanning angle of ±1.7°) and 41.17° (right-looking with azimuth scanning angle of ±1.6°), which shows that the effects (for same azimuth resolution) of the processing enhancements are much more obvious as the range is much longer, namely, the looking angle is larger. This is consistent with the theoretical analysis. Especially for small looking angle, it brings very minor improvement of PSLR and even very minor deteriorations of both azimuth resolution and ISLR, although all these changes are negligible.

### 5.2. Ultra-Fine Stripmap Product and the Dual-Receive Imbalance

**Figure 9:**
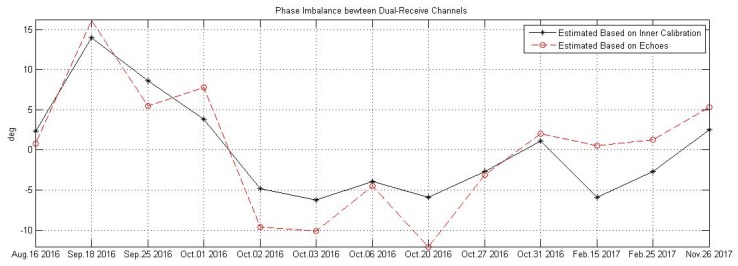
The amplitude and phase imbalance between dual-receive channels of ultra-fine stripmap mode of GF-3 monitored at the commissioning phase.

**Figure 10:**
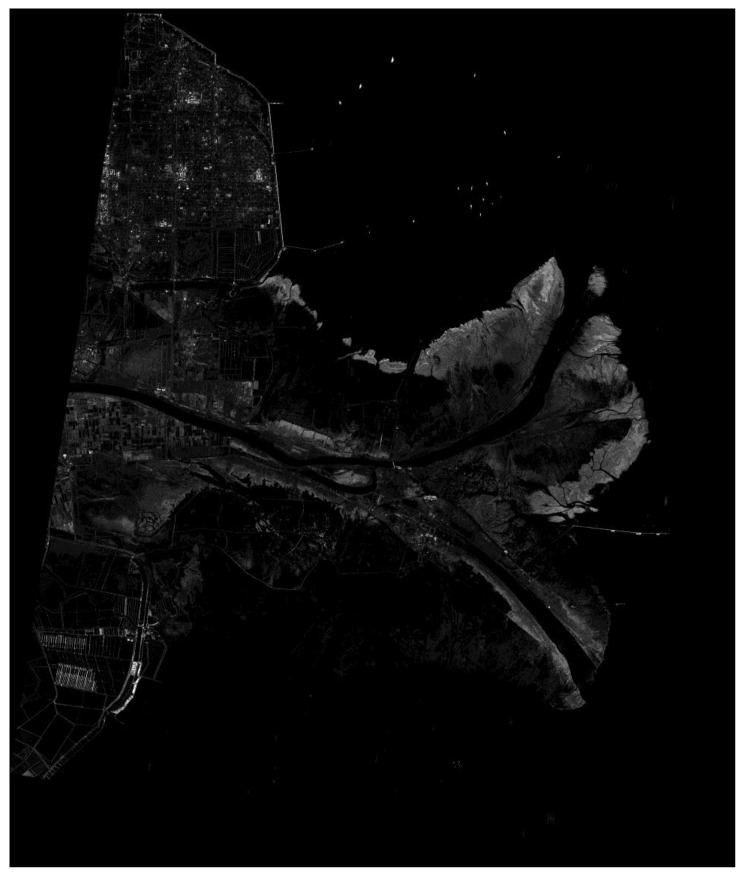
The GF-3 ultra-fine stripmap imagery of the estuary of Huanghe River China (acquired at 15 February 2017).

Since GF-3 has been launched, the amplitude and the phase imbalance between dual-receive channels of ultra-fine mode were monitored continuously. Figure 9 presents the phase imbalance estimated based both on the echo signal and the inner calibration signal before or after echo data acquiring, which shows that the phase imbalance was varying in about −15° to +15°, and the trends of the variety with time indicated by inner calibration signal and echo signal are highly consistent with each other. The imbalance estimating basing on echo signal is only necessary when the inner calibration signal was not acquired correctly. Figure 10 gives the imagery of the estuary of Huanghe River China acquired by ultra-fine stripmap mode of GF-3 with incidence angle from 48.539322° to 49.791040° at 15 February 2017.

### 5.3. Quad Polarization Stripmap Product and Quad-Pol Imbalance

**Figure 11:**
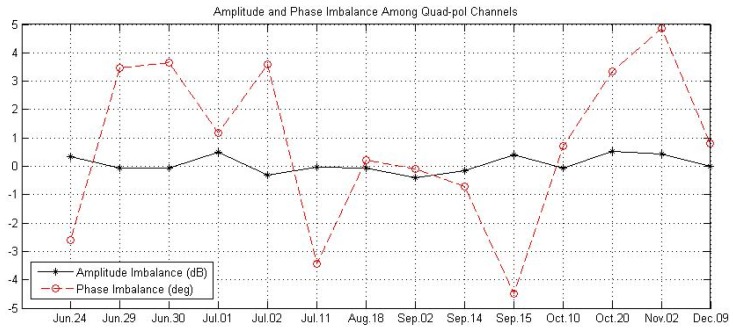
The amplitude and phase imbalance among quad-pol channels of full polarization stripmap mode of GF-3 after being in operational application.

**Figure 12:**
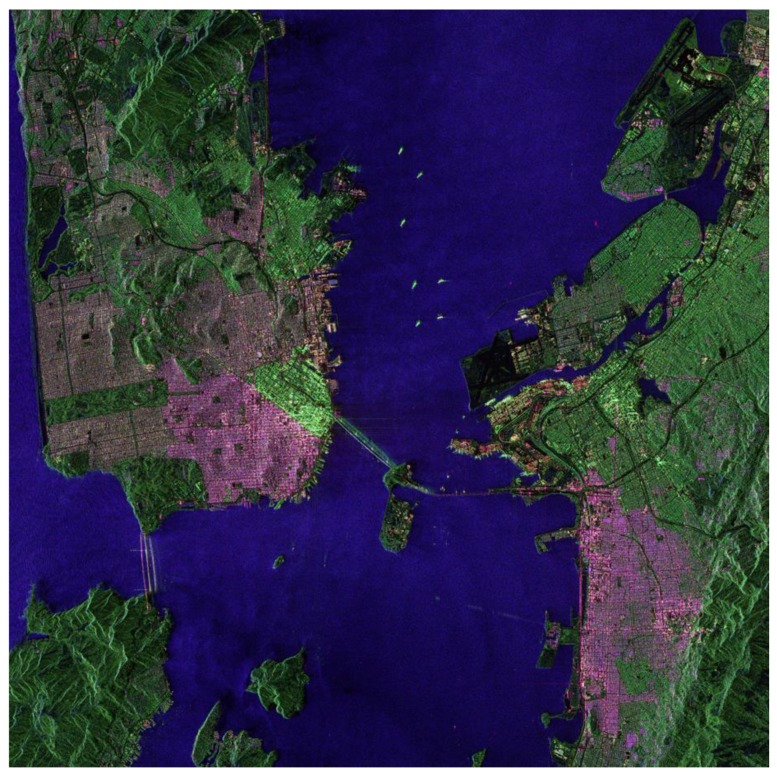
The GF-3 quad-pol stripmap Pseudo-colored imagery of San Francisco of America (acquired at 15 September 2017).

After the GF-3 satellite was in operational application, the amplitude and phase imbalance among quad-pol channels of all quad polarization modes (quad-pol stripmap mode, wide quad-pol stripmap mode and wave mode) were monitored from June 2017 to December 2017 for about half a year as shown in Figure 11. The variety of amplitude imbalance was less than 0.5 dB and the variety of phase imbalance was less than 10° which achieve the predicted performance, if the quad-pol product imagery has been calibrated by both the inner calibration and the external calibration, namely the values of “DoFPInnerImbalanceComp” and “DoFPCalibration” in meta-data file are both “1”. Figure 12 gives the imagery of San Francisco of America acquired by quad-pol stripmap mode of GF-3.

### 5.4. ScanSAR Scalloping Suppressing

**Figure 13:**
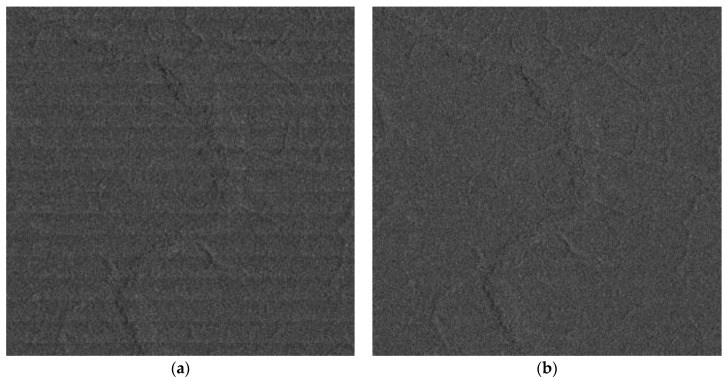
The GF-3 narrow scanSAR imagery (**a**) before de-scalloping and (**b**) after de-scalloping of rainforest of Brazil (acquired at 25 February 2017).

Figure 13 gives the GF-3 narrow scanSAR imagery of rainforest of Brazil acquired at 25 February 2017. Based on the de-scalloping processing mentioned in Section 3.4, the scalloping was reduced from 2 dB to 0.5 dB, which make the scanSAR image quality achieve an obvious enhancement.

## 6. Conclusions

After four-month payload commissioning phase and two-month application commissioning phase, the quality of the imagery products for all observing modes generated by GF3DP achieves the expected performance, some aspects are even better than the predicted indexes [1]. Since January 2017, the GF-3 satellite has officially been in operational application. The image products for 12 observing modes, which were generated in the history can be inquired by all customers on the website of http://www.cresda.com, can be ordered for free by all its primary users such as the State Oceanic Administration (SOA), the China Meteorological Administration (CMA), the Ministry of Civil Affairs, and the Ministry of Water Resources.

## Figures and Tables

**Table 1:** Product Formats of GF-3.

Level	Type	Format
Level 1A	Single look complex imagery in slant range geometry (SLC)	TIFF + RPC
Level 1B	Single or multiple looks intensity imagery in slant range geometry (S/MLI)	TIFF + RPC
Level 2	Single or multiple looks intensity imagery geocoded (SGC)	GeoTIFF

**Table 2:** Primary parameters for one performance test experiment of spotlight mode of GF-3.

Parameter	Value
Observing Mode	Spotlight
Polarization	VV
Incidence Angle	47.197573°–47.674581°
Resolution	1.0 m (A) × 1.0 m (GR ^1^)
Swath	10 km (A) × 10 km (GR)
Relative Radiometric Accuracy	1.0 dB (in one scene)
Absolute Geolocation Accuracy ^2^	10 m (Maximum Value)
Peak Sidelobe Ratio	−22.0 dB (A) × −22.0 dB (R)
Integral Sidelobe Ratio	−15.0 dB (A) × −15.0 dB (R)

^1^ GR means ground range; ^2^ the absolute geolocation accuracy was given by all three corner reflectors.

**Table 3:** Results before the spotlight processor applied the enhancements.

		Resolution (m)	PSLR (dB)	ISLR (dB)
CR1	Slant Range	0.6734	−23.0007	−20.0332
	Azimuth	0.9084	−19.5094	−18.0584
CR2	Slant Range	0.6762	−22.346	−20.3446
	Azimuth	0.9131	−19.8803	−18.2674
CR3	Slant Range	0.6707	−21.9888	−20.0494
	Azimuth	0.8197	−21.1818	−17.6952
Mean Value	Slant Range	0.6734	−22.4452	−20.1424
	Azimuth	0.8804	−20.1905	−18.007

**Table 4:** Results after spotlight processor applied the enhancements.

		Resolution (m)	PSLR (dB)	ISLR (dB)
CR1	Slant Range	0.6734	−23.006	−20.0523
	Azimuth	0.8735	−22.3987	−18.7712
CR2	Slant Range	0.6762	−22.3719	−20.3598
	Azimuth	0.8739	−22.9284	−19.0292
CR3	Slant Range	0.6707	−22.1987	−20.1118
	Azimuth	0.8627	−22.6787	−18.9657
Mean Value	Slant Range	0.6734	−22.5255	−20.1746
	Azimuth	0.8700	−22.6686	−18.922

**Table 5:** Performances ^1^ of azimuth compression at different looking angles of spotlight imaging.

	Center Look Angle		Resolution (m)	PSLR (dB)	ISLR (dB)
1	22.37°	N	0.8165	−22.9521	−18.8756
	Left-looking	Y	0.8219	−22.9906	−18.6104
2	36.52°	N	0.8189	−19.7917	−17.608
	Right-looking	Y	0.8199	−22.9741	−18.8724
3	41.17°	N	0.8804	−20.1905	−18.007
	Right-looking	Y	0.8700	−22.6686	−18.922

^1^ Results of the three groups in Table 5 are obtained under the same weighting parameters, each one is the expectation of the results for 3 or 4 corner reflectors.

**Figure A1:**
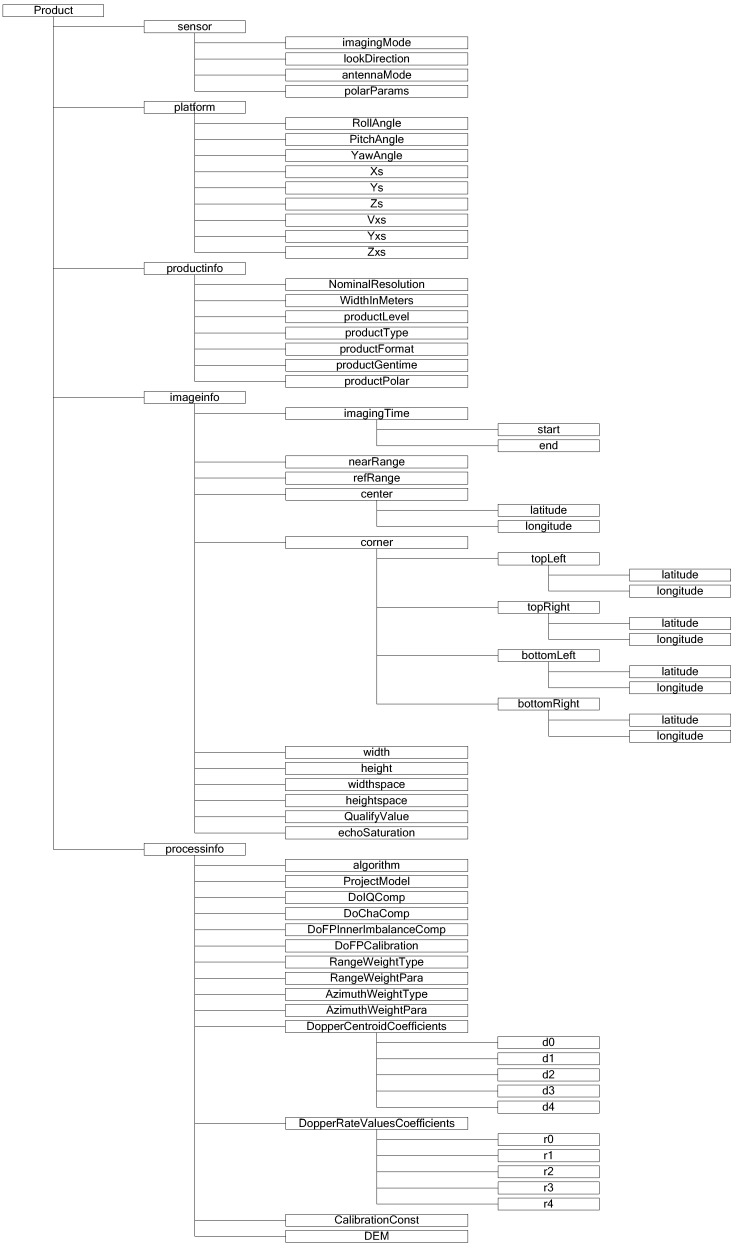
An overview of the XML meta-data structure for GF-3 products.

## Appendix A

Several expressions about the motion compensation of the spotlight processor in Section 3.1 are briefly indicated in this appendix. Three primary expressions mentioned here are Δ*R_sr_*, ΔΦ*_c_* and Φ_3*m*_.

Δ*R_sr_* describes the non-linear and non-uniform movement of the spaceborne SAR relative to the virtual rotating point of sliding spotlight imaging, which can be expressed as:

(A1) $$\Delta R_{sr}(t)=R_{sr}(t)-R_{esr}(t)$$

where *t* is the azimuth time or slow time, *R_sr_*(*t*) is the real range history between the antenna phase center of the spaceborne SAR and the virtual rotating point within the imaging time, and *R_esr_*(*t*) is the equivalent hyperbola of *R_sr_*(*t*) which is generally used in SAR imaging.

ΔΦ*_c_* describes the remnant cubic phase error within the accumulating time (namely, the synthetic aperture) after the range history average error correction has been performed, which is approximately azimuth-space-invariant for GF-3 spotlight imaging parameters. According to the principle of stationary phase (POSP), the remnant cubic phase error in time domain can be easily corrected in Doppler domain, which can be expressed as:

(A2) $$\Delta \Phi_{c}(f)\approx \frac{4\pi \Delta p_{3}f^{3}}{\lambda f_{Rst}^{3}}$$

where *f* is the frequency in Doppler domain, *λ* is the wave length, *f_Rst_* is the Doppler rate of the echo signal for a certain target on the Earth. As above, the non-linear and non-uniform movement of the spaceborne SAR relative to the target can be expressed as Δ*R_st_*(*t*). Δ*p*_3_ is the third order coefficient of Taylor’s polynomial for Δ*R_st_*(*t*) − Δ*R_sr_*(*t*) near center of the imaging time.

Φ_3*m*_ is the modified third factor of chirp scaling algorithm, which completes the azimuth compression of sliding spotlight data being azimuth prefiltered and the compensation of the remnant cubic phase error. It is expressed as:

(A3) $$\begin{array}{cc} \Phi_{3m}\left(\tau ,f\right) & =\exp\left\{-j\frac{2\pi }{\lambda }c\tau \left[1-\sin\varphi_{ref}\sqrt{1-{\left(\frac{\lambda f}{2\upsilon }\right)}^{2}}\right]+j\frac{\pi f^{2}}{f_{Rsr}}+j\Delta \Phi_{c}\right\}\\ & \cdot \exp\left\{j\left[\Theta_{1}\left(f\right)+\Theta_{2}\left(f;r\right)\right]\right\} \end{array}$$

where *τ* is the range time or fast time, *c* is the speed of light, *φ_ref_* is the equivalent squint angle at referenced slant range, *ν* is the equivalent velocity at range gate where the target is located, *f_Rsr_* is the fictitious Doppler rate for the virtual rotating point, Θ_1_(*f*) and Θ_2_(*f*;*r*) are the remnant phase error of CS algorithm [18].

## Appendix B

Figure A1 presents an overview of the meta-data of GF-3 product, for which the overall and detailed descriptions have been given in the GF-3 products description handbook [17]. At the first level of the meta-data, it consists of five primary records as “sensor”, “platform”, “productinfo”, “imageinfo”, and “processinfo”. There will be brief descriptions for each primary record and its critical subrecords.

The “sensor” is about the parameters those are properties of the SAR sensor from the auxiliary data, which consists of “imagingMode” (the value can be “SL” for spotlight mode, “UFS” for ultra-fine stripmap mode, “FSI” for fine stripmap mode, “FSII” for wide fine stripmap mode, “SS” for standard stripmap mode, “NSC” for narrow scanSAR mode, “WSC” for wide scanSAR mode, “GLO” for global observation mode, “QPSI” for quad-pol stripmap mode, “QPSII” for wide quad-pol stripmap mode, “WAV” for wave mode and “EXT” for expanded incidence angle mode), “lookDirection” (the value can be “R” for right-looking direction and “L” for left-looking direction), “antennaMode” (the value can be “S” for single antenna receive phase center and “D” for double antenna receive phase centers), “polarParams” (the value can be HH, VV, HHHV, VHVV and AHV) and etc.

The “platform” is about the parameters those describe the moving status of the satellite platform, which consists of the position vector, the velocity vector and attitude parameters (in yaw, pitch and roll direction) of the satellite at the center imaging time.

The “productinfo” is about the parameters those describe the type of output product, which consists of “NominalResolution”, “WidthInMeters”, “productLevel”, “productType”, “productFormat”, “productGentime” and “productPolar”.

The “imageinfo” is about the parameters those describe the final output imagery, which consists of the latitude and longitude information about the four corners and the center of the imagery, the size of the imagery, the pixel space of imagery, the qualify value, the ratio of the echo with saturation and etc.

The “processinfo” is about the parameters those describe the processing method using in product generation process, which consists of the information about the “algorithm” type (the value can be “CS”, “DCS”, “ECS” and etc.), the projecting method, the channel calibration flag, the weighting parameters, the radiometric calibration constant, the Doppler parameters and etc.
